# CMV and EBV targets recognized by tumor-infiltrating B lymphocytes in pancreatic cancer and brain tumors

**DOI:** 10.1038/s41598-018-34710-2

**Published:** 2018-11-20

**Authors:** Qingda Meng, Davide Valentini, Martin Rao, Ernest Dodoo, Markus Maeurer

**Affiliations:** 1 Division of Therapeutic Immunology (TIM), Department of Laboratory Medicine (LABMED), Karolinska Institutet, Stockholm, Sweden; 2 Centre for Allogeneic Stem Cell Transplantation (CAST), Karolinska University Hospital Huddinge, Stockholm, Sweden; 3 Department of Neurosurgery, Karolinska University Hospital, Stockholm, Sweden

## Abstract

Targeted antiviral immune responses to the widespread human pathogens cytomegalovirus (CMV) and Epstein-Barr virus (EBV) play a pivotal role in determining immune fitness. We show here for the first time that tumor-infiltrating B cell (TIB)- derived immunoglobulin G (IgG) from patients with pancreatic cancer or glioblastoma have unique anti-CMV/EBV immune recognition patterns compared to serum IgG. There is also great heterogeneity between patients, as well as between serum and TIB-IgG, while some viral targets elicited strongly both T-cell and IgG reactivity in tumor infiltrating T- and B-cells. These observations suggest that the anti-CMV/EBV humoral immune response *in situ* is highly unique and can be instrumental in developing next-generation immuno-biomarkers in addition to supplementing cellular therapy strategies for personalized cancer therapy targeting CMV or EBV in the tumor microenvironment.

## Introduction

Immune responses directed against cytomegalovirus (CMV) and Epstein-Barr virus (EBV) are indicative of immuno-physiological fitness of an individual^1–3^. The involvement of CMV in modulating cellular immune responses in cancer has been reported in humans as well as in preclinical studies^4–6^, while EBV-driven immune responses appear to be implicated in (EBV+ ) nasopharyngeal carcinoma (NPC), hematological malignancies^7–9^ and gastric carcinoma^10,11^. Most clinical studies have focused on the T-cell response to CMV or EBV and the current concept of immune protection suggests that intact memory CD8^+^ and CD4^+^ T helper 1 (Th1) response patterns contribute to long-term protection against viremia^2,12,13^.

Anti-CMV or anti-EBV specific T-cell responses have been shown to be biologically and clinically relevant in active immunotherapy: activation of CMV pp65-specific T cells in patients with glioblastoma (GBM), via a cell-based vaccination strategy, led to remarkable reduction in disease burden and increased patient survival^14^, while adoptive transfer of *ex vivo*-expanded autologous T cells specific for EBV antigens has been shown to produce clinically relevant effects in patients with loco-regional NPC^15^. Thus, T-cell responses targeting CMV/EBV can be harnessed for therapy.

Tumor-infiltrating lymphocytes (TIL) may confer tumor regression patients with solid cancers^16–20^. Although TIL largely comprise T-cell populations, a substantial proportion of these cells consist of tumor-infiltrating B cells (TIB), which contribute to the regulation of intratumoral immune responses^21,22^. TIB have the capacity to produce effector cytokines i.e. tumor necrosis factor alpha (TNF-α), interferon gamma (IFN-γ) or interleukin 21 (IL-21), in addition to processing and presenting antigens to orchestrate the local cellular immune response^21^. TIB produce also antibodies that maybe directed against tumor-associated antigens (TAAs) or persistent viral antigens such as those derived from CMV and EBV in the tumor microenvironment.

CMV- and EBV-directed humoral immune responses have not yet been studied in the context of advanced human cancers in great detail, although some studies suggested that CMV or EBV antibody-mediated immune reactivity is associate with well-preserved general antiviral immunity^7,23^. In the present study, we evaluated antibody responses from tumor-infiltrating B cells (TIB) to epitopes derived from CMV pp65 as well as EBV proteins (EBNA-1, EBNA-3a, LMP-2 and BMLF1) using a peptide microarray platform and compared the viral protein-directed immunoreactivity of serum immunoglobulin gamma (IgG) to TIB-derived IgG (TIB-IgG). EBNA-1, EBNA-3a and LMP-2 are latency-associated EBV proteins, while BMLF1 is expressed during the lytic cycle of the virus. These antigens also contain immunogenic CD4+ T-cell epitopes, particularly EBNA1^24^. BMLF1 also induces CD8+ T-cell responses in healthy individuals as well in individuals with EBV-driven infectious mononucleosis. Intact immune responses to these proteins may reflect i) optimal control of viral replication, thus the host’s ability to keep the pathogen under immune surveillance and ii) the capacity to mount protective immune responses in general^1,25^.

CMV pp65 is a major T-cell antigen which is frequently used as a surrogate target of cellular immune responses gauging ‘immune fitness’ in *in vitro* cell-based assays; uncompromised T-cell reactivity to CMV pp65 may imply good control of viral replication^26^. Besides the observation that CMV pp65- directed T cells may target GBM cells^27^, it also serves as a target for antibody responses^28–30^. Thus, CMVpp65, as well as proteins from the lytic and latent cycles of EBV replication represent viable candidates to mine for B-cell reactivity and to map antibody recognition profiles.

CMV-specific T-cells have been described in tumor (melanoma) lesions^31^; we describe here to our knowledge for the first time qualitative and quantitative differences in viral target recognition of tumor-associated B-cells in patients with pancreas cancer and GBM.

## Materials and Methods

### Patient description

Serum samples were obtained from 3 patients with pancreatic cancer and 12 patients with brain tumors, while TIB samples were available for 18 patients with cancer (9 patients with pancreatic cancer and 9 with brain tumors). This study was approved by the Regional Ethics Review Board (Regionala etikprövningsnämnden) at Karolinska Institutet, Sweden (EPN: 2013/576-31, CNS tumors and 2013/977-31/1 and 2013/1332-31/3, pancreatic cancer). In addition, written informed consent was obtained from the patients prior to initiation of study. Methods were performed in accordance with the relevant guidelines and regulations. The clinical characteristics of the patients with cancer are provided in Table 1.

**Table 1:** Clinical characteristics of patients.

Patient ID	Age	Gender	Histology	Antibody source for peptide microarray
PanTT*24	81	F	Pancreatic ductal adenocarcinoma	TIB
PanTT26	61	M	Pancreatic ductal adenocarcinoma	Serum
PanTT32	48	F	Pancreatic ductal adenocarcinoma	TIB and serum
PanTT39	62	F	Distal bile duct adenocarcinoma	TIB
PanTT41	74	M	Pancreatic ductal adenocarcinoma	TIB
PanTT43	69	M	Pancreatic ductal adenocarcinoma	TIB
PanTT47	69	M	Duodenal adenocarcinoma	TIB
PanTT53	66	M	Pancreatic ductal adenocarcinoma	TIB
PanTT55	71	M	Pancreatic ductal adenocarcinoma	TIB
PanTT68	81	M	Pancreatic ductal adenocarcinoma	TIB
PanTT77	68	M	Pancreatic ductal adenocarcinoma	Serum
BRT*1	70	M	Glioblastoma	TIB and serum
BRT2	32	F	Pleomorphic xanthoastrocytoma	TIB and serum
BRT3	59	F	Glioblastoma	TIB
BRT4	70	F	Glioblastoma	TIB
BRT5	32	M	Astrocytoma	TIB
BRT6	59	M	Glioblastoma	TIB
BRT7	66	M	Renal cell carcinoma metastasis	TIB
BRT8	56	M	Glioblastoma	TIB
BRT9	16	M	Glioblastoma	TIB
BRT10	52	F	Glioblastoma	Serum
BRT11	43	M	Glioblastoma	Serum
BRT12	48	M	Oligodendroglioma	Serum
BRT13	52	M	Glioblastoma	Serum
BRT14	33	F	Astrocytoma	Serum
BRT15	62	F	Glioblastoma	Serum
BRT16	63	F	Glioblastoma	Serum
BRT17	52	M	Glioblastoma	Serum
BRT18	71	F	Glioblastoma	Serum
BRT19	67	F	Glioblastoma	Serum

^*^PanTT, Pancreatic cancer tumor tissue; BRT, Brain tumor tissue.

### Preparation of PBMCs and serum from whole blood

Whole blood of patients with cancer was treated with Ficoll-Hypaque solution (GE Healthcare, Uppsala, Sweden) to isolate PBMCs as well as serum. The cells were washed twice prior to storage at −176 °C (liquid nitrogen), while the sera were aliquoted and stored at −80 °C for later analysis.

### Peptide microarrays

A total of 33 clinical samples (serum, n = 15 and TIB supernatant, n = 18) were incubated in three 12-plex peptide microarray chips embedded with 12-mer peptides from the following viral proteins (the UniProt ID is provided in parentheses): CMV pp65 (P06725), EBV EBNA1 (P03211), EBV EBNA3 (P12977), EBV LMP2 (P13285), and EBV BMLF1 (Q04360). The 12-mer peptides were selected to entirely cover each protein sequence with an 11-amino acid overlap. A total of 3067 peptides spanned the five viral proteins, with each peptide represented seven times (seven technical replicates) on the microarray chip. The antibodies used in the peptide microarrays for detecting IgG responses to peptides was Alexa Fluor® 647 AffiniPure Goat Anti-Human IgG, Fcγ Fragment Specific, reconstituted to 1 mg/ml (Jackson ImmunoResearch, West Grove, PA; cat# 109-605-098). After incubation, the microarrays were scanned with a NimbleGen MS200 scanner (Roche Applied Science, Penzberg, Germany) and signal was extracted with an internal Roche software called SlideViewer. The signal intensity of each spot was measured, following which a recognition data matrix was produced.

### Statistical and data analysis

The data was preprocessed using the RMA-background correction method^32^, followed by normalization using *loess* spatial correction^33^ and log2 transformation. Since comparison between arrays or array groups are not within the scope of this study, no between-array normalization was performed. The intensities of the repeated peptides were averaged (by sample) within each group comprising all peptides belonging to the same viral protein. Coefficients of variation (CV = σ/μ) of intensities were also computed for each peptide across its technical repetitions per biological sample. Considering that high dispersion of these signal values could be a possible indication of spot artifacts or anomalies, peptide repetitions with large coefficient of variation (>1) were identified, flagged and the corresponding spots checked manually. After averaging, cleaning and applying QC measures, a panel of 2882 unique peptides was obtained for each chamber. Robust zeta scores were computed (*z* = *(x − median(x))*/*mad(x)*) separately for each sample. Peptides that showed very high IgG reactivity were identified by setting a detection cut-off corresponding to 3 times the standard deviation of each sample’s zeta values.

### Generation of TILs and TIB cultures

TILs from patients with pancreatic cancer or brain tumor were isolated and expanded *in vitro* with the addition of IL-2, IL-15 and IL-21 as previously described^34,35^. Briefly, fresh tumor tissue was cut into 1–2 mm^3^ pieces using a sterile scalpel, washed twice with cold PBS and cultured in 24-well plates containing T-cell medium ((Cellgro GMP-grade serum-free medium (CellGenix, Freiburg, Germany) with 10% pooled human AB serum (Innovative Research, Novi, MI), supplemented with recombinant human cytokines (Prospec, Ness-Ziona, Israel): IL-2 (1000IU/ml), IL-15 (10 ng/ml) and rhIL-21 (10 ng/ml)). Medium replenishment was carried out as necessary. Irradiated allogeneic PBMCs (55 Gy) were used as feeder cells and added at a ratio of 1:10 (feeders:TIL) after seven days of culture initiation. TIL were transferred to six-well plates upon achieving >70% confluence in the 24-well culture plates. Further expansion of TILs was performed in G-Rex flasks (Wilson Wolf, St. Paul, MN) with 30 ng/ml OKT3 (BioLegend, San Diego, CA) and irradiated allogeneic feeder cells added at a ratio of 1:5.

### TIB cultures

Fresh tumor tissue from patients with pancreatic cancer or brain tumor was cut into 1–2 mm^3^ pieces using a sterile scalpel. Each fragment was cultured in 24-well plates, with each well containing 1 ml TIB medium ((70% Cellgro GMP-grade serum-free medium (CellGenix, Freiburg, Germany), 20% B95-8 supernatant containing EBV virus (filtered with 0.22um filter), 10% FBS, penicillin (100 IU/mL), streptomycin (100 μg/mL) (Life Technologies, Carlsbad, CA) and amphotericin B (2.5 mg/L) (Sigma-Aldrich, St Louis, MO)). After 7 days of culture, half the culture volume was replaced with TIB medium (without the B95-8 supernatant).

### Antibody supernatant from TIB

TIB cell lines were washed twice with PBS to remove FBS, which contains bovine antibodies, to reduce false positive detection. The cells were then cultured in 1 ml of Cellgro GMP-grade serum-free medium (without the addition of human AB serum and FBS) in individual wells of 24-well tissue culture plates (cell suspension density of 1 × 10^6^ cells/ml/well). After five days of culture, the supernatants were harvested and stored at −80 °C for peptide microarray studies.

### Evaluation of TIL and PBMC immunoreactivity to CMV and EBV peptides

Based on the signal intensity of the peptide microarray analysis and statistical significance, the top five viral peptides (regardless of pathogen) recognized by each TIB IgG sample were selected for chemical synthesis (Peptides and Elephants, Henningsdorf, Germany). Next, 1.0 × 10^5^ TILs were cultured in 200 μl of T-cell medium in the presence of 5 μg/ml of the individual viral peptides in round-bottom 96-well tissue culture plates. Cells in T-cell medium alone served as negative control while cells incubated with 30 ng/mL of the anti-human CD3 antibody (clone OKT3, BioLegend, San Diego, CA) were used as positive control to induce maximal stimulation of the TCR. The cells were incubated for three days at 37 °C with 5% CO_2_, following which culture supernatants were harvested for measuring IFN-γ production using a standard sandwich enzyme-linked immunosorbent assay (ELISA) kit (Mabtech, Stockholm, Sweden). The peptide-specific IFN-γ production is reported after subtracting values from the negative control (T-cell medium only) to reflect the readout as IFN-γ production (in pg/3 days/1.0 × 10^5^ T cells).

## Results

### Antibody reactivity to peptides spanning whole viral proteins

**Figure 1:**
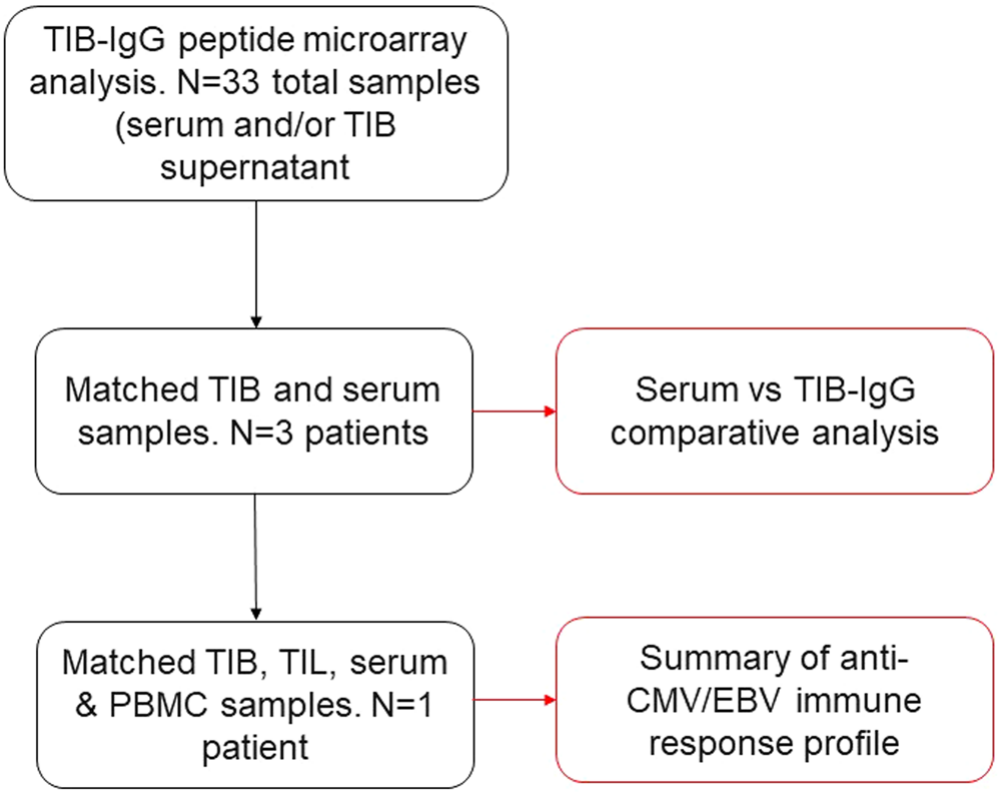
Schematic representation of sample inclusion in experiments and data analysis. Peptide microarrays was performed with 33 samples from 30 patients, comprising a combination of tumor-infiltrating B lymphocyte (TIB) supernatants and serum samples. Following analysis of the resulting data, matching serum and TIB supernatant samples from 3 patients were used for comparative analysis of CMV/EBV peptide recogniton patterns in the tumor versus the periphery. We studied the matching TIL as well as PBMC responses to the top five viral peptides based on peptide microarray data, which lead to selecting a matched set of samples from only a single patient.

**Figure 2:**
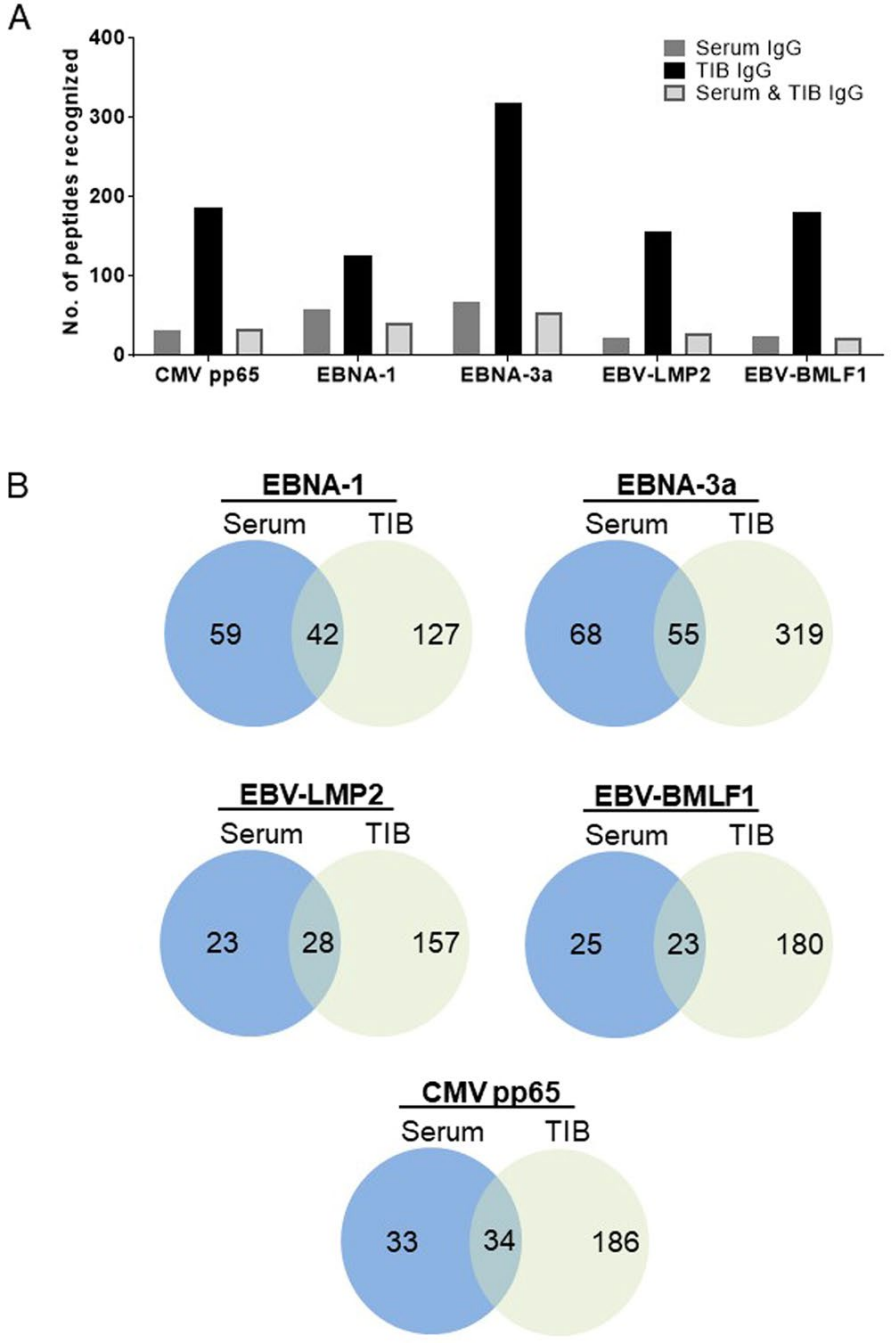
Overview of CMV and EBV peptides recognized by serum and/or TIB-IgG. (**A**) Serum (n = 15) as well as TIB supernatant (n = 18) samples (totaling to 33 independent biological samples) which were tested on the peptide microarray chips containing peptides spanning CMV pp65 and EBV proteins (EBNA-1, EBNA-3a, LMP2 and BMLF-1) were analyzed, and the total number of viral peptides recognized by the respective sample types are presented. TIB-IgG showed the highest degree of diversity in terms of recognition pattern, while serum IgG a much more restricted profile. Some viral peptides exhibited shared recognition by serum and TIB-IgG and at a level comparable to that recognized by serum IgG alone. (**B**) Venn diagrams showing the number of uniquely recognized viral peptides by serum and/or TIB-IgG at higher resolution. Of note, peptides which were recognized by serum and TIB-IgG (shared targets) were not recognized by serum IgG or TIB-IgG alone (individual targets).

A diagram depicting the number of patient samples used in this study is shown in Fig. 1. Antibody responses to CMV and EBV were probed using high-content peptide microarrays encompassing the whole sequence of the following EBV proteins: EBNA1, EBNA3, LMP2 and BMLF1, as well as CMV pp65. Serum as well as TIB supernatant samples from 15 and 18 patients with cancer, respectively, were used as the source of the primary binding antibody to probe for specific IgG-reactive viral protein epitopes. The final sets of data were produced after highly stringent statistical analysis, as mentioned in the Materials and Methods section. TIB-IgG displayed broad recognition of a large panel of epitopes from all five viral test proteins, while serum IgG showed a more restricted epitope recognition pattern (Fig. 2A).

EBNA3 exhibited the highest number of epitopes recognized in all three analysis categories (serum IgG only = 68; TIB IgG only = 319; serum & TIB IgG = 55). EBNA1 ranked second in terms of the number of epitopes recognized by serum IgG alone (n = 59), followed by CMV pp65 (n = 34), EBV-BMLF1 (n = 25) and EBV-LMP2 (n = 23) (Fig. 2B). As compared to EBNA3, EBV-BMLF1 displayed the lowest number of epitopes recognized by both serum and TIB-IgG (n = 23). Using the Chi-square test, we found that in general, more epitopes were recognized by serum IgG compared to shared recognition by serum & TIB IgG (p < 0.001). However, the number of peptides derived from EBV-BMLF1 (p > 0.85) and CMVpp65 (p = 1) recognized by serum vs serum & TIB IgG were quite similar. Of note, the individual peptides that were recognized by serum and TIB-IgG (shared targets) were not separately recognized by serum or TIB-IgG (individual targets). A heat map of the viral peptides that were most frequently recognized by serum and/or TIB-IgG is provided in Supplementary Figure 1.

**Figure 3:**
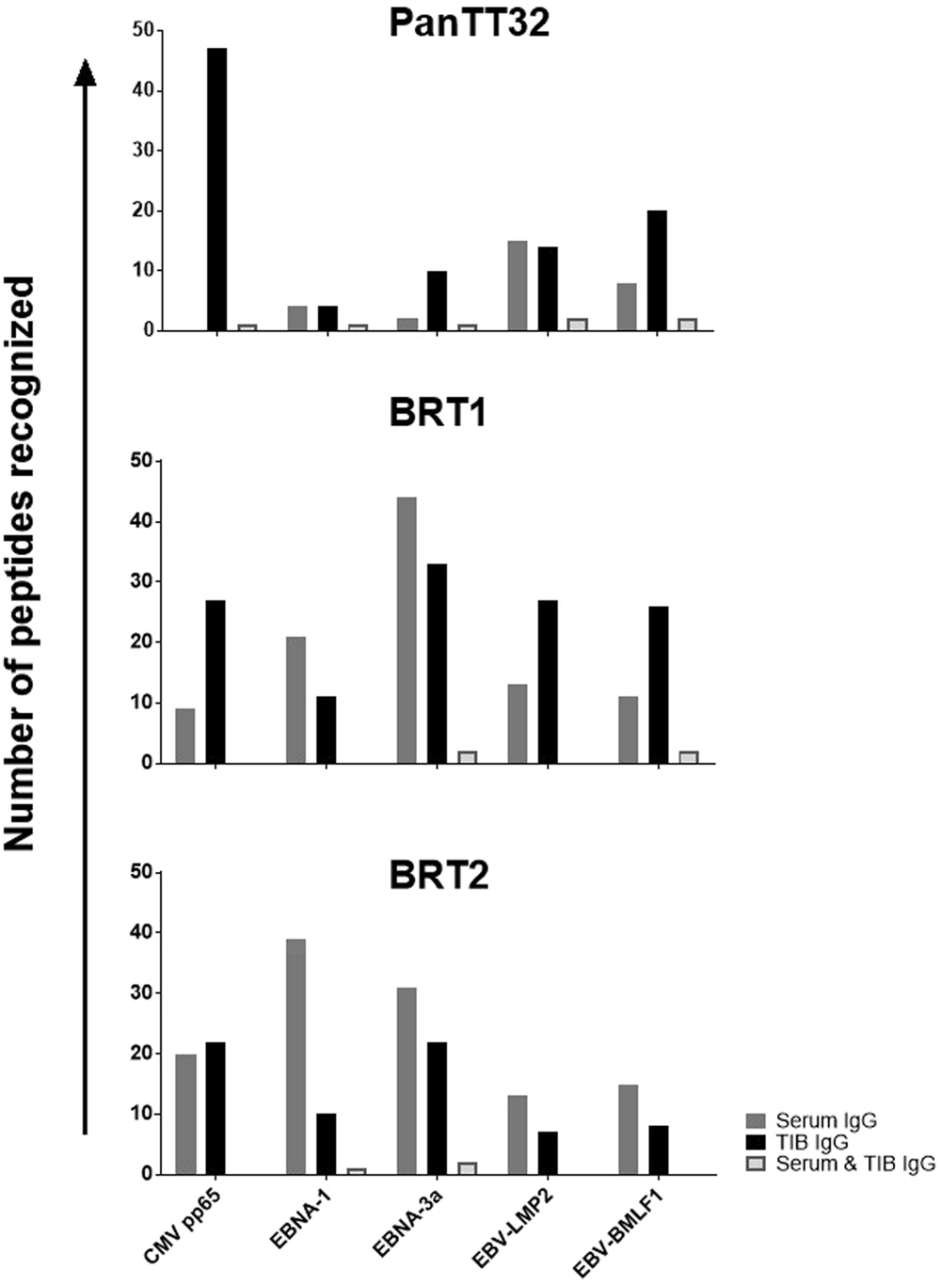
Individualized IgG recognition pattern of viral targets across patients. We performed a more detailed analysis in matching serum and TIB supernatant samples from three patients. Each patient displayed a unique IgG recoginition pattern of CMV and EBV epitopes. PanTT32 showed the strongest recognition of CMV pp65 peptides by TIB-IgG, and exhibited a shared recognition of all the viral peptides tested (from CMV and EBV proteins). BRT1 and BRT2 showed a much more pronounced recognition of viral proteins by serum IgG, albeit the shared recognition pattern (serum and TIB-IgG) was weaker compared to PanTT32.

15 serum samples and 18 TIB supernatant samples were analyzed from patients with cancer, three samples were obtained from matched donors, which allowed us to analyze the intra-patient viral epitope recognition profile of the serum versus TIB-IgG. The recognition of viral epitopes was heterogeneous across the three patients. PanTT32 exhibited a strong recognition of CMV pp65 epitopes exclusively by TIB IgG, shared recognition of any of the five viral protein targets was minimal (Fig. 3). The strongest serum IgG recognition of viral epitopes in this patient was observed for EBV-LMP2 peptides, with a similar number of targets uniquely recognized by TIB IgG. Analysis of immune responses from a patient with glioblastoma (BRT1) showed a very different viral target recognition profile, with a more pronounced recognition of viral peptides by serum IgG as compared to PanTT32. Similarly, TIB IgG from BRT1 also exhibited strong recognition of peptides derived from CMV pp65, EBNA3, EBV-LMP2 and EBV-BMLF1, and to a lesser extent, EBNA1. Furthermore, serum and TIB IgG from BRT1 displayed shared recognition of only EBNA3 and EBV-BMLF1 peptides but not the remaining three viral proteins. Analysis of immune responses from another patient with glioblastoma (BRT2) showed a different pattern: the viral test targets showed more robust serum IgG reactivity as compared to TIB IgG. As for shared recognition of viral peptides by serum and TIB IgG from BRT2, immune responses were exclusively observed for EBNA1 and EBNA3 peptides. In summary, the recognition profile of CMV as well as EBV protein targets by circulating (serum) or TIB-IgG shows a high degree of heterogeneity between patients, while shared recognition of viral antigens by serum and TIB-IgG appears to be uncommon – reflecting a differential antigen recognition patterns in the periphery as opposed to the tumor microenvironment.

### T-cell reactivity to viral peptides highly recognized by TIB IgG

**Figure 4:**
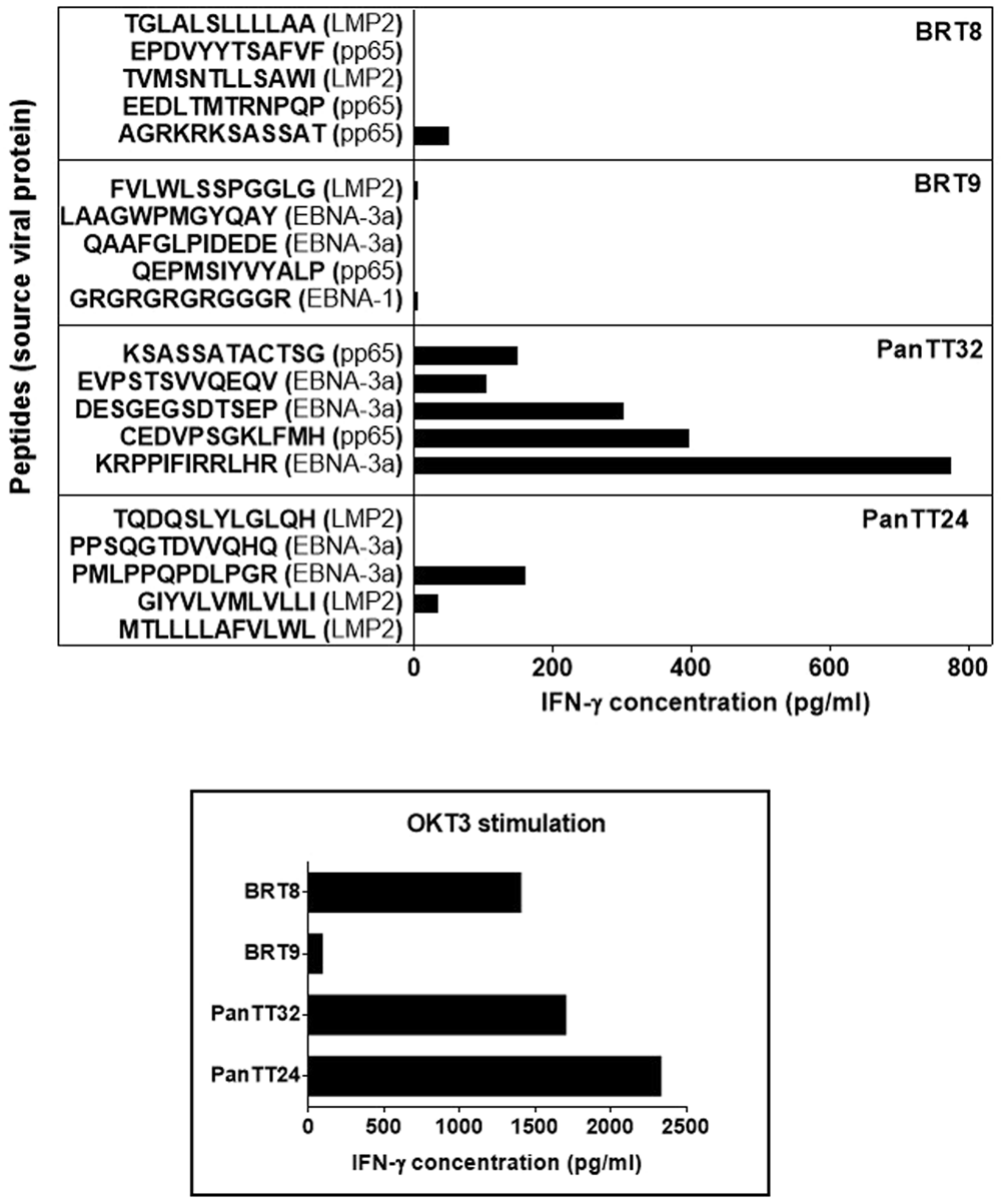
TIL responses to viral peptides recognized by TIB-IgG. The top five viral peptides that induced the most robust IgG responses (stongest signal intensity) on the peptide microarrays were selected for determining whether they also contained T-cell epitopes. Selected peptides were chemically synthesized and co-cultured with the corresponding TIL from four patients with cancer (BRT8, BRT9, PanTT32 and PanTT24) over a 3-day period. The anti-human CD3 monoclonal antibody OKT3 was used a positive control for T-cell receptor (TCR) stimulation, thus as an indicator of T-cell functionality. Supernatants were then harvested for measuring IFN-γ production by ELISA, the concentration values of which are expressed as pg/10e5 T cells. TIL from patient PanTT32 showed a robust responses to two CMV pp65 peptides and three EBV EBNA-3a peptides, while TIL from BRT9 exhibited the weakest immune reactivity to any of the viral peptides tested, probably due to reduced overall functionality – marked by poor stimulation by OKT3. PanTT24 TIL displayed strong anti-EBV responses, while TIL from patient BRT8 elicited measurable responses to a single CMV pp65 peptide. Note that other immune readouts, i.e. production of different cytokines, other than IFN-γ or T-cell proliferation have not been captured in the current assay readout.

Based on the peptide microarray readout, we next selected four patients (PanTT32, PanTT24, BRT8 and BRT9) whose TIB-IgG displayed the strongest (highest signal intensity) responses to the CMV and EBV peptides tested. The top five peptides recognized by TIB-IgG in supernatants from these patients were chemically synthesized and co-cultured with TIL from the respective patients in order to test for the presence of T-cell reactivity by measuring IFN-γ production. OKT3 stimulation (anti-human CD3 antibody) was used as a positive control for T-cell functionality. T-cell reactivity was very pronounced in the TIL cell line obtained from patient PanTT32 (pancreatic cancer), who displayed strong responses to stimulation with two CMV pp65 peptides and three EBV EBNA-3a peptides (Fig. 4) defined by IFN-γ production. The most prominent response was observed with the EBV EBNA-3a peptide KRPPIFIRRLHR (776 pg IFNγ /10e5 T cells) in PanTT32 TIL, while two CMV pp65 peptides (KSASSATACTSG and CEDVPSGKLFMH) also elicited IFN-γ production (150 pg and 396 pg IFNγ /10e5 T cells, respectively). In addition to inducing strong IgG as well as TIL responses, the peptide KRPPIFIRRLHR was detected by IgG in 4/18 (22%) TIB supernatant samples (Supplementary Figure 1). TIL from patient BRT9 displayed the weakest reactivity, also to OKT3 stimulation (94 pg/ml) with only basal IFN-γ production in response to the EBV EBNA-1 peptide GRGRGRGRGGGR (5 pg IFNγ /10e5 T cells) and the EBV LMP2 peptide FVLWLSSPGGLG (5 pg IFNγ/10e5 T- cells). No response was observed for CMV pp65 peptide QEPMSIYVYALP in TIL from patient BRT9. A similar pattern of weak IFN-γ production in response to viral peptides was seen in BRT8 TIL, albeit with strong responsiveness to OKT3 (positive control). A single CMV pp65 peptide (AGRKRKSASSAT) elicited measurable cellular reactivity in this TIL line (50 pg IFNγ/10e5 T cells). We also observed a strong IFN-γ response to the EBV EBNA-3a peptide PMLPPQPDLPGR (160 pg/10e5 T- cells) in TILs from patient PanTT24. Thus, we concluded that CMV and EBV peptides contain shared IgG and TIL epitopes recognized in the tumor microenvironment.

### Strong CMV-directed immune responses in patient PanTT32

Based on the above data, we intended to form a summary of the four different readouts of immune responses seen with the biological samples from patients with cancer: TIB-IgG, TIL, serum IgG and immune reactivity in PBMCs. We had all four samples matched for only a single patient, namely PanTT32 (Table 2). The top five TIB-IgG-reactive viral peptides were ranked in sequential order (1 → 5) with respect to the strength of recognition (signal intensity) observed in the peptide microarray experiments. The TIL and PBMC responses to each of these peptides were then ranked in accordance to the amount of IFN-γ production induced in the *in vitro* immune reactivity assays (strongest → weakest). The serum IgG data is not included as a separate column in the table since only a single CMV pp65 peptide (CEDVPSGKLFMH) fell into this category. However, the same peptide also induced strong IFN-γ responses from TIL (rank 2) as well as PBMC (rank 1) and was the fourth most strongly recognized target by TIB-IgG (indicated by the red box), suggesting that strong immune response directed against the CMV pp65 peptide CEDVPSGKLFMH may be a robust indicator of humoral and cellular immune reactivity.

**Table 2:** Summary of immune response readout for patient PanTT32.

PanTT32	Peptide	Rank in PM output	Intratumoral T-cell response		Peripheral T-cell response	
Source protein			TIL IFN-γ (pg/ml)	Rank	PBMC IFN-γ (pg/ml)	Rank
CMVpp65	KSASSATACTSG	1	150	4	14	3
EBNA3	EVPSTSVVQEQV	2	105	5	1	5
EBNA3	DESGEGSDTSEP	3	302	3	22	2
**CMVpp65**	**CEDVPSGKLFMH**	**4**	**396**	**2**	**220**	**1**
EBNA3	KRPPIFIRRLHR	5	776	1	1	4

^*^The bold indicates the CMV pp65 peptide which induced strong T-cell as well as IgG responses in patient PanTT32. PM = peptide microarray.

## Discussion

To the best of our knowledge, this is the first study to show that CMV- and EBV-directed humoral immune responses exist in human tumor tissue based on the viral epitope reactivity of TIB-IgG produced *ex vivo*. TIB IgG responses are more diverse as opposed to the more focused serum IgG response. Therefore, antiviral antibody responses that are observed in tumor tissue appear to be directed against a more broad panel of viral epitopes. A recognition pattern similar to broad neoantigen-specific T-cell responses – which when activated/mobilized - may result in tumor regression and improved survival in patients with advanced cancer^36–38^. Furthermore, it was recently shown that a higher number of B cells (CD20^+^ lymphocytes) infiltrating cutaneous melanoma lesions, based on immune-histological staining, is correlated with improved survival^39^.

CMV infection and GBM have been very closely linked due to several lines of clinical evidence: (i) presence of CMV DNA in GBM tumors^40,41^; (ii) reduction of viral load by valganciclovir treatment contributing to GBM regression^42^ (iii) specific recognition and killing of GBM cells by CMV pp65-specific T cells^27^ and (iv) tumor regression and survival benefit observed in patients with GBM following treatment with CMV pp65-pulsed dendritic-cell vaccine^14^. One study in a preclinical model of brain cancer showed that CMV promotes the activation of a focal adhesion kinase, necessary for glioma cell integrin-mediated cell migration and cancer cell invasiveness^43^. Although the clinical studies mentioned above provide evidence (in part) for an active role for CMV-directed T-cell responses in GBM, ours is the first effort which shows that antibody reactivity to CMV targets can also be found in the tumor microenvironment in humans.

To our knowledge, no study has yet to link EBV infection and pancreatic cancer or GBM directly. However, considering that CMV infection and GBM share a potential association, we decided to also investigate whether patients with pancreatic cancer, as a ‘control cohort’, also display EBV/CMV-directed humoral immune responses. EBV-specific antibodies have been found to be an important hit for type 1 diabetes mellitus in a high-throughput assay mining for potential immunologically relevant targets^44^, supporting the notion that presence of EBV-directed humoral immune responses may be associated with pancreatic disease. However, it has to be noted that the patients with pancreatic cancer or GBM relevant to this study were not tested for CMV/EBV infection at admission, i.e. prior to surgical removal of the tumor lesion.

The quality and quantity of antibody reactivity to viral epitopes is biologically relevant. In addition to binding viral particles, B cells may directly take up and process antibody-bound, virus-infected apoptotic tumor cells^45^. B cells may also facilitate the enhanced uptake of antibody-bound apoptotic material by infiltrating macrophages and dendritic cells, which can in turn amplify local and systemic T-cell responses against non-viral targets^46,47^. Not mutually exclusive, the presence of IgG specifically targeting epitopes of CMV and EBV may reflect the ‘immunological fitness’ of patients with cancer and may, therefore, serve as a reliable screening tool to guide immunotherapies. For instance, strong T-cell responses to CMV and EBV antigens, represented by IFN-γ production, are able to predict survival for patients with pulmonary tuberculosis, and represent a possible surrogate target of immune fitness in chronic (bacterial) disease characterized immune dysregulation^1^.

The broad recognition of viral targets by TIB-associated IgG antibodies may represent a unique imprint of immune reactivity in the tumor microenvironment. Erkes and colleagues showed that CMV-specific T cells can infiltrate established melanoma lesions (in a preclinical model) while retaining their functional properties, regardless of PD-1 expression^5^.Thus, virus-specific adaptive immune cells that are present in cancer lesions may well have a biologically relevant role in promoting anti-tumor responses. Furthermore, since CMV proteins are present in GBM tissue^41^, targeted immune responses against relevant viral epitopes by intratumoral (TIB-associated) IgG molecules warrants further exploration. Recent data showed intra-tumoral polarization of T cells, driven by bacterial species in patients with colorectal cancer^48^. Common viral pathogens, such as EBV or CMV, may impact in a similar fashion on the shaping of the ‘*milieu interne*’ of cancer lesions, i.e. on the quality and breadth of the *in situ* anti-cancer directed immune response.

Antibody responses may serve to sustain T-cell responses in patients with cancer. CD4^+^ and CD8^+^ T-cell epitopes are likely to be recognized by antibodies and present a valuable diagnostic platform in patients with cancer mediated by the tumor-associated antigen (TAA) NY-ESO-1^49–51^. We describe in the current report that TIL respond to CMV and EBV peptides - which were initially identified by TIB-IgG. This response seems to be unique for each individual, although the infectious agent is likely to be identical. We show in the present study that the CMV pp65 peptide CEDVPSGKLFMH appears to be a strong T-cell target while inducing measurable humoral immune reactivity. This peptide may be a good candidate for gauging ‘immune fitness’ in immune cell-based therapeutic approaches. Not mutually exclusive, TIB-derived (CMV or EBV-specific) IgG may also be indicative of ‘cross-reacting’ antibodies recognizing tumor-associated targets, in the form of wildtype or mutant antigens, as suggested by the testing of clinically relevant outcomes (survival) of patients with pancreatic cancer: tumor neoantigen ‘quality’, defined by T-cell recognition, was increased by homology of human (cancer) targets to infectious disease-derived peptide targets^52^. Epitopes from CMV or EBV-reactive TIB may provide such qualities, i.e. to select for stronger and long-lasting humoral and cellular immune responses against cancer – associated antigens and common (viral) pathogens.

Intra-tumoral B cells may not only promote productive immune responses defined by the production of pro-inflammatory cytokines, such as TNF-α, yet they may also produce anti-inflammatory, ‘tolerizing’ cytokines. The production of transforming growth factor beta (TGF-β) and IL-10 by regulatory B cells can inhibit the differentiation and activity of tumor-directed CD8+ T cells, Th1 cells and NK cells while promoting the expansion of FoxP3^hi^ CD4+ regulatory T cells by MHC class II-mediated presentation of tolerizing epitopes^46^. Some plasma cells can also produce antibodies which, in fact, trigger the further development of myeloid-derived suppressor cells (MDSCs) that impede anti-tumor responses *in situ*. In contrast, B effector type 1 (Be1) cells which produce pro-inflammatory cytokines i.e. IL-12, IFN-γ can prime Th1- and CD8+ T-cell activity. IL-12/IL-18, elaborated by inflammatory immune cells *in situ*, can also drive IFN-γ production in Be1 cells^53,54^. Formation of tertiary lymphoid organs (TLO) in the tumor, also involving B-cell populations in addition to T-cell subsets in patients with lung cancer, is associated with survival benefit^55^. TIB themselves can present antigens to promote anti-tumor immunity while, independent of the MHC pathway, engage CD27 on the surface of T cells to amplify anti-tumor responses^56^. Jiang *et al*.^57^ reported that herpes simplex virus 1 (HSV-1)-specific IgG can accumulate in neuronal tissue (transgerminal ganglion), consistent with protection against herpesvirus-driven disease. Taken together, the presence of antibody-secreting plasma cells in GBM and pancreatic cancer shed light on the possibility that these immunoglobulins may orchestrate local immune responses in both modalities, i.e. pro- and anti-tumor, and warrant further investigation for elucidating their exact role(s) in tumor-directed immune responses.

Productive humoral immune responses to EBV may hold clinical significance in EBV+ cancers, i.e. NPC, gastric carcinoma^10^, Burkitt’s lymphoma, B-cell lymphomas (Hodgkin’s and non-Hodgkin’s), B cell-associated post-transplant lymphoproliferative disorder (PTLD)^58^ and several T cell-associated malignancies^59^. We are not aware of studies demonstrating EBV-specific B cells in these tumor lesions, although saliva from patients with NPC were found to contain EBNA-1-specific IgA, possibly rising from TIBs – in conjunction with detection of membrane-associated EBNA-1 expressed in NPC cells^60^. Infiltration of T-bet+ (Th1) and CD20+ (B cells) in gastric carcinoma has also been associated with survival benefit^61^, lending support to the notion that EBV-specific B cells are indeed a constitutive immune cell subset in TIL. TIB appear to play an important role in mediating control of hepatocellular carcinoma (HCC), in addition to TIL expressing granzyme B and IFN-γ^62^. The authors also observed that human IgG were bound to HCC cells in tumor tissue samples, presenting evidence that TIB may produce tumor-directed antibodies *in situ*.

An important drawback in the present study is the combined use of unmatched serum and TIB IgG from the patients, except for three patients for whom we obtained matching samples. Inter-individual (and inter-sample) variability is certainly a factor to consider when studying immune responses in humans, particularly patients with cancer. The immunological milieu in the tumor microenvironment (and systemically) may differ from one patient to another i.e. antigen turnover, T-cell recognition patterns, mutational load, intra-tumoral cytokine levels, etc.^63,64^. However, we observed a generally more focused recognition of viral targets by serum IgG as compared to TIB-derived IgG, which suggested that this is possibly a common pattern in TIL from patients with pancreatic or brain cancer. Another technical difficulty faced was the inability to produce a stable TIB-cell line from some patients for generating TIB-derived IgG; for these patients, only the serum was available for the peptide microarray studies. We do acknowledge these shortcomings in the study, which must be considered when drawing conclusions of the implications of the results.

Our laboratory recently published an article on cellular and humoral immune responses to EBV/CMV antigens in patients with pancreatic or brain cancer^65^. In this report, we showed that patients with glioblastoma who presented with high titres of anti-CMVpp65 circulating IgG experienced increased survival. Furthermore, patients with pancreatic cancer generally exhibited a much higher anti-CMV IgG titre as well as anti-viral cellular immune responses (IFN-γ production) in blood compared to patients with glioblastoma. Although survival data would be clinically relevant here, we do not have this information for the patients whose samples were tested for IgG reactivity in the present study. However, we have been able to observe that strong cellular immune responses (represented by IFN-γ production in whole blood assays) to EBV and CMV antigens in patients with pancreatic cancer post-surgery is concomitant with a survival benefit (Supplementary Figure 2). This is clinically relevant to IgG recognition of immunogenic targets since cellular immune activation by certain antigens can occur in concert with increased humoral immune responses^66–68^, in addition to being indispensable for controlling chronic infections^69,70^. Anti-CMV or anti-EBV directed cellular immune cells targeting viral epitopes *in situ* may also be used as recipient cells for anti-tumor directed T-cell receptors, or chimeric antigen receptors (CARs) expressed as transgenes, combining anti-viral and anti-tumor directed targeting in a single immune effector cell population.

In summary, the current study contributes to the existing body of knowledge that CMV/EBV-directed humoral immune responses have a role in mediating clinically relevant immune responses in cancer, particularly pancreatic cancer and GBM. Most of the EBV-transformed B-cell lines exhibited a CD20+ CD27- HLA-DR+ phenotype (Supplementary Figure 3) – yet would need to undergo more detailed examination concerning the BCR (B-cell receptor) repertoire, complemented by in *situ* immuno-histological analysis in future studies. These insights may be used in the development of host-directed therapies or immunodiagnostic platforms, taking advantage of shaping the tumor microenvironment.

## Conclusion

Highly specific antibody as well as T-cell reactivity to CMV and EBV epitopes in patients with pancreatic cancer or glioblastoma indicate that antiviral immune responses may constitute an integral component of the *in situ* host defense in cancer. Anti-CMV/EBV immune responses in cancer are heterogeneous; they are both patient- and disease-specific. Harnessing these responses may serve as a good starting point to develop adjunctive immune-based interventions to broaden the treatment options available to patients diagnosed with advanced cancers.

## Electronic supplementary material

Supplementary data set Figures S1-S3
