# Supplementary material for: CMV and EBV targets recognized by tumor-infiltrating B lymphocytes in pancreatic cancer and brain tumors
Source: Sci Rep. 2018 Nov 20;8:17079. doi: 10.1038/s41598-018-34710-2 (PMC6244284; doi:10.1038/s41598-018-34710-2)
Supplement: Supplementary file 1 — Supplementary data set Figures S1-S3 [file 41598_2018_34710_MOESM1_ESM.pdf]

SUPPLEMENTARY ONLINE MATERIAL

Supplementary Figures S1-S3

CMV and EBV targets recognized by tumor-infiltrating B lymphocytes in pancreatic cancer and brain tumors

Qingda Meng, Davide Valentini, Martin Rao, Ernest Dodoo and Markus Maeurer

EBNA1

Serum only		TIB only	
CIGCKGTHGGTG	12	RGRGGGRPGAPG	4
GCKGTHGGTGAG	11	FEYHQEGGPDGE	4
GGGAGAGGAGGA	11	LYNLRRGTALAI	3
PPRRPPPPGRRPF	10	GAIEQGPADDPG	3
LRALLARSHVER	10	GGRGRGGSGGRG	3
RGGSRERARGRG	9	VCYFMVFLQTHI	3
VRRPQKRPSCIG	8	RGRGGSGGRGRG	3
AGGAGGAGAGGG	8		
GRRPFFHPVGEA	7		
DEGGDGDEGEEG	5		
RALLARSHVERT	5		
ARGGSRERARGR	5		
HIFAEVLKDAIK	5		
DGEPDVPPGAIE	4		
ADDPGEGPSTGP	4		
GAGAGGAGGAGG	4		
RGRGRGEKRPRS	4		
GPSTGPRGQGDG	3		
KDLVMTKPAPTC	3		
GAGGGAGAGGGA	3		
RGQGDGGRRKKG	3		
RGRGEKRPRSPS	3		
GDEGGDGDEGEE	3		
PVGEADYFEYHQ	2		
PPWFPPMVEGAA	2		
PGRRPFFHPVGE	2		
GRGRGEKRPRSP	2		
GRPGAPGGSGSG	2		
GGAGGAGGAGAG	2		
LSRLPFGMAPGP	2		
RPSCIGCKGTHG	2		
PGAPGGSGSGPR	2		
GGGAGAGGGAGG	2		
AGGAGAGGGAGG	2		

Serum & TIB	Serum	TIB
ADYFEYHQEGGP	1	1
AEGLRALLARSH	8	2
AGAGAGGAGAGG	6	1
AGAGGGGRGRGG	12	1
AGGAGAGGGGRG	3	2
AGGGAGAGGAGG	10	2
DPGEGPSTGPRG	1	2
DYFEYHQEGGPD	1	1
EADYFEYHQEGG	1	1
EGGDGDEGEEGQ	1	1
EVLKDAIKDLVM	6	1
FMVFLQTHIFAE	4	2
GAGGGGRGRGGS	7	1
GEPDVPPGAIEQ	2	2
GGAGAGGGGRGR	5	1
GGAGGAGGAGGA	11	1
GGRPGAPGGSGS	11	1
GGRRKKGGWFGK	1	1
GGSGSGPRHRDG	8	2
GGTGAGAGAGGA	2	1
GMAPGPGPQPGP	4	1
GPGNGLGEKGDT	2	1
GPRHRDGVRRPQ	13	2
GQGGSNPKFENI	3	1
GRGGSGGRRGRG	2	1
GRGRGGSGGRRG	1	1
GRGRGRGRGRGG	8	1
GRRKKGGWFGKH	6	1
HGGTGAGAGAGG	11	1
KRPRSPSSQSSS	1	1
LRRGTALAIPQC	1	1
PDVPPGAIEQGP	1	3
PFFHPVGEADYF	2	2
PRGQGDGGRRKK	8	3
PSTGPRGQGDGG	1	3
RPQKRPSCIGCK	10	3
RRPFFHPVGEAD	5	1
SGPQRRGGDNHG	3	2
THGGTGAGAGAG	2	2
VERTTDEGTWVA	1	1
VFVYGGSKTSLY	1	1
VPPGAIEQGPAD	13	1

EBV-LMP2

Serum only		TIB only	
LLILAYRRRWRR	10	LLLASALIAGGS	3
DDSSQHIYEEAG	9	LPPPPYSPRDDS	3
YYCLTLESEERP	9	PSPGGDPDGYDG	3
GVIRCCRYCCYY	7	LTLAAALALLAS	3
PPNDEERESNEE	7	LLMLLWTLVVLL	3
GLPPPPYSPRDD	6	AAGGLQGIYVLV	3
FLLMLLWTLVVL	5	FCMLLLIVAGIL	3
FLIGFALFGVIR	4	NLTTMFLLMLLW	3
LTAVVTFFAICL	3		
QHDGNDGLPPPP	3		
SCPLSKILLARL	2		
WRIEDPPFNSLL	2		
LLTLAAALALLA	2		

Serum & TIB	Serum	TIB
AALALLASLILG	1	1
ACVLVLIVDAVL	2	2
APYLFWLAAIAA	4	1
AVASSYAAAQRK	1	1
DPYWGNGDRHSD	4	2
FAICLTWRIEDP	3	3
GGIMFLACVLVL	4	1
GGLQGIYVLVML	6	1
GILFILAILTEW	4	1
GIYVLVMLVLLI	9	2
ILLARLFLYALA	1	2
IVAPYLFWLAAI	1	1
KILLARLFLYAL	1	1
LAAAGGLQGIYV	2	1
LASLILGTLNLT	2	2
LLLLAAVASSYA	1	1
MCLGGLLTMVAG	5	1
MTLLLLAFVLWL	1	3
MVPMGAGPPSPG	9	1
NLFCMLLLIVAG	11	1
PSASGSSGNTPT	2	2
RWRRLTVCGGIM	2	1
SLILGTLNLTTM	1	1
SSPGGLGTLGAA	2	1
TFFAICLTWRIE	2	1
WLAAIAASCFTA	6	2
VMLVLLILAYRR	1	2
VPMGAGPPSPGG	3	1

EBV-BMLF1

Serum only		TIB only	
TRKQARQERSQR	11	ILTKLKELAPCF	3
NCRRRHDEVE	10	TLREFFTKSTNK	3
ETGNSSYTRGHK	9	NKPWFDMSLVKP	3
PSQRLSRTSSIS	8	VPRAPRSPRAPR	3
PTSASFVIPRKK	6	LSRTSSISSNED	3
HRSPLCRDEDEK	5	LASLTLEPIQDP	3
LAVAAHPEIGAW	5	LEETIFWLQEIT	3
KITFVTLPSPLA	4	EDEDPTPAHAIP	3
RGAGRSTRKQAR	3	WLQEITYHGDLP	3
CTDESYGKRRHL	2	RPLPNKPWFDMS	3
QAIQGLCTLV	2	ANCRRRHDEVEE	3
FLGHYIKNYNPG	2	PLCLLAAYAAVA	3
SRGAGRSTRKQA	2	ARQERSQRPLPN	3

Serum & TIB	Serum	TIB
AHAIPARPSSVV	7	2
AHPEIGAWQKVQ	7	1
AMLEETIFWLQE	11	2
APAYINCRRR	2	2
AVSDTNTDCDLD	1	2
CRSASCSRLVRA	2	1
DARMQAIQGL	7	3
DPFLQSMLAVAA	2	2
EITYHGDLPLAP	3	1
EKEETGNSSYTR	9	1
GEVHGCTDESYG	4	3
GHYIKNYNPGTL	6	1
LCRDEDEKEETG	4	1
LDPMEGSEEHST	1	1
NEDPAESHILEL	1	2
NRATRGPRSESR	1	1
PGTGSLGFFVP	1	2
QLFYITCATARQ	1	1
RKKWDLQDKTVT	7	1
SMLAVAAHPEIG	1	1
SYVKQPLCLLAA	1	1
TDCDLDPMEGSE	7	2
TSASFVIPRKKW	1	2

EBNA3

Serum only		TIB only	
VPPVPATQPQYF	13	EFLGFLQRTDLS	4
QGMAYPLHEQHG	12	ERPVYPKPVRPA	4
SQYFDLPLTQPI	12	KRPPIFIRRLHR	4
RPRPRTPEWPVQ	11	SAGTFKLPRCTP	4
SAPARPMLPPQP	9	ISNTEMYIMYAM	4
PAAHFLHQPPME	9	LLRTEGEHVEGA	3
GTDVVQHQLDAL	9	GTPRPPVPKPRP	3
TIHLAAQGMAYP	8	WPVQEEGGQDAT	3
EFPDLQDPPDEA	7	PGVFSDGRVACA	3
QGEGGQNVTGPE	5	YQAYSSWMYSYT	3
WENVLIELSDSS	5	IHLAAQGMAYPL	3
TTPTFVHLQATL	4	PINHGAPAAHFL	3
QSCNPRYSIFFD	4	AFGLPIDEDESG	3
SVGNIVQSCNPR	4	CTPGDRQWLYVQ	3
LRTLLQAIGAAA	4	RTEQGKEVLEKA	3
ATSHGSAQVPEP	3	QSDETATSHGSA	3
AAFGLPIDEDES	3	KPRPEVPQSDET	3
VRPGVAQSQYFD	3	HRSLTKIWEEVL	3
LARLRAEAQVKQ	3	LPLIQPISQGAP	3
EGPLVPEQWMFP	3	IPLTEPINQGAS	3
CQDDEFPDLQDP	3	YMAIHRSLTKIW	3
LTEPINQGASAA	3		
ADVVRAPGVPAM	3		
LIELSDSSSEKE	3		
QAPEWPVQEEGG	3		
DETATSHGSAQV	3		
QPINHGAPAAHF	3		
PVPTVALERPVY	2		
MDKDRPGPPALD	2		
EEVPSTSVVQEQ	2		
EPINQGASAAHF	2		
SVVQEQVSAGDW	2		
VVVSAVVHMCQD	2		
EDTESDGDDEDL	2		
ASMGPVPPVPAT	2		
HGSAQVPEPPTI	2		

Serum & TIB	Serum	TIB
AAQGMAYPLHEQ	1	2
AGTFKLPRCTPG	1	1
AIGAAATRIDTR	3	1
AKWRLQTLAAGW	1	1
AQGMAYPLHEQH	3	1
AQVKQASVEVQP	1	3
DIPLTEPINQGA	2	2
DQFFGSQISNTE	10	1
DRPGPPALDDNM	11	1
DTSEPCEALDLS	1	4
EDESGEGSDTSE	2	1
EKEAEDAHLEPA	3	3
ETREESEDTESD	3	1
FDLPLTQPINHG	4	1
GAPAAHFLHQPP	3	1
GNIVQSCNPRYS	2	1
GPIVRPWEPSLT	2	1
GPLVPEQQMFPG	1	3
GQDATEVLDLSI	5	1
GVPAMQPQYFDL	3	3
HLQATLGCTGGR	9	2
ILRRFPLDLRTL	1	1
IRQAIRDRRRNP	1	2
KEAEDAHLEPAQ	6	1
LHGLNHPGVPVS	6	1
LRTEGEHVEGAT	1	1
LVPEQQMFPGAP	4	1
MEGPLVPEQWMF	2	1
MFQGAPPSQGTD	1	1
MGPVPPVPATQP	1	1
MRAGKRTEQGKE	1	1
PGPPALDDNMEE	1	1
PGVPAMQPQYFD	4	2
PLVPEQQMFPGA	1	1
PPQLTQVSPQQP	4	1
PVPATQPQYFDI	1	1
PVRPQHMPVEPV	3	3
QASVEVQPPQLT	9	1
QGPGETSGIRRA	2	2
QPMEGPLVPEQW	1	1
QSQYFDLPLTQP	6	3
RARERWRPAPWT	5	2
RAYGIDLLRTEG	2	1
RPCPQAPEWPVQ	2	4
RTDLSYIKSFVS	1	1
RTPEWPVQGEGG	7	1
SIFFDYMAIHRS	6	2
SRGGPKVKRPPI	1	1
TREESEDTESDG	9	3
VAQSQYFDLPLT	5	1
WMFPGAALSQSV	1	1
VPTVALERPVYP	1	3
YFDLPLIQPISQ	3	1
YGIDLLRTEGEH	5	3
YVQSSVGNIVQS	14	2

CMV pp65

Serum only		TIB only	
LVCSMENTRATK	13	NIPSINVHHYPS	4
KEPDVYYTSAFV	10	EPDVYYTSAFVF	4
VYYTSAFVFPTK	9	KSIPGLSISGNL	4
YRIFAELEGVWQ	8	TRATKMQVIGDQ	3
QTGIHVRVSQPS	8	LEYRHTWDRHDE	3
TYFTGSEVENVS	7	HNPTGRSICPSQ	3
AVIHASGKQMWQ	5	YVVALPLKMLNI	3
SIPGLSISGNLL	4	SGVMTRGRLKAE	3
IRETVELRQYDP	4	EDLTMTRNPQPF	3
PVADAVIHASGK	4	PAVFTWPPWQAG	3
HYPSAAERKHRH	4	AWTRQQNQWKEP	3
KISHIMLDVAFT	3	AGRKRKSASSAT	3
GGAMAGASTSAG	3	LKAVFSRGDTPV	3
GDNQLQVQHTYF	2	VQHTYFTGSEVE	3
VFPTKDVALRHV	2	ENTRATKMQVIG	3
ISHIMLDVAFTS	2		
RHLPVADAVIHA	2		
SATACTSGVMTR	2		
AELEGVWQPAAQ	2		

Serum & TIB	Serum	TIB
AGILARNLVPMV	5	1
CAHELVCSMENT	2	1
CEDVPSGKLFMH	2	1
CPKSIPGLSISG	4	2
CPSQEPMSIYVY	8	1
DEELVTTERKTP	1	1
DNQLQVQHTYFT	3	1
EMISVLGPISGH	5	1
FGLLCPKSIPGL	2	1
FPTKDVALRHVV	4	3
FWDANDIYRIFA	8	2
GHVLKAVFSRGD	1	1
HDEGAAQGDDDV	11	1
HEHFGLLCPKSI	1	1
IHVRVSQPSLIL	1	2
IYRIFAELEGVW	7	5
IYVYALPLKMLN	1	1
KAVFSRGDTPVL	3	1
KLFMHVTLGSDV	3	1
KSASSATACTSG	3	1
LEGVWQPAAQPK	6	1
LFFFDIDLLLQR	6	1
LSISGNLLMNGQ	1	2
MISVLGPISGHV	8	1
MWQARLTVSGLA	2	1
NLKYQEFFWDAN	2	1
NQLQVQHTYFTG	7	2
PDVYYTSAFVFP	1	1
PTKDVALRHVVC	3	4
TRQQNQWKEPDV	3	3
TVSGLAWTRQQN	8	2
WPPWQAGILARN	7	1
VQAIRETVELRQ	1	1
WQPAAQPKRRRH	1	1

Frequency:

Low — High

Supplementary Figure 1. **Heat map of the most frequently recognized viral peptides.** CMV and EBV peptides which were recognized in at least 15% of samples (2 serum samples or 3 TIB supernatant samples) were tabulated and ranked according to frequency of recognition by IgG. As for the shared recognition profiles, a viral peptide which was recognized at least once by both serum and TIB-IgG was included in the table. The color codes symbolize a gradient, ranging from low (white) to high frequency recognition (red), while the individual numbers indicate the number of samples in which a measurable signal (positive response/recognition) was detected.

Supplementary Figure 2

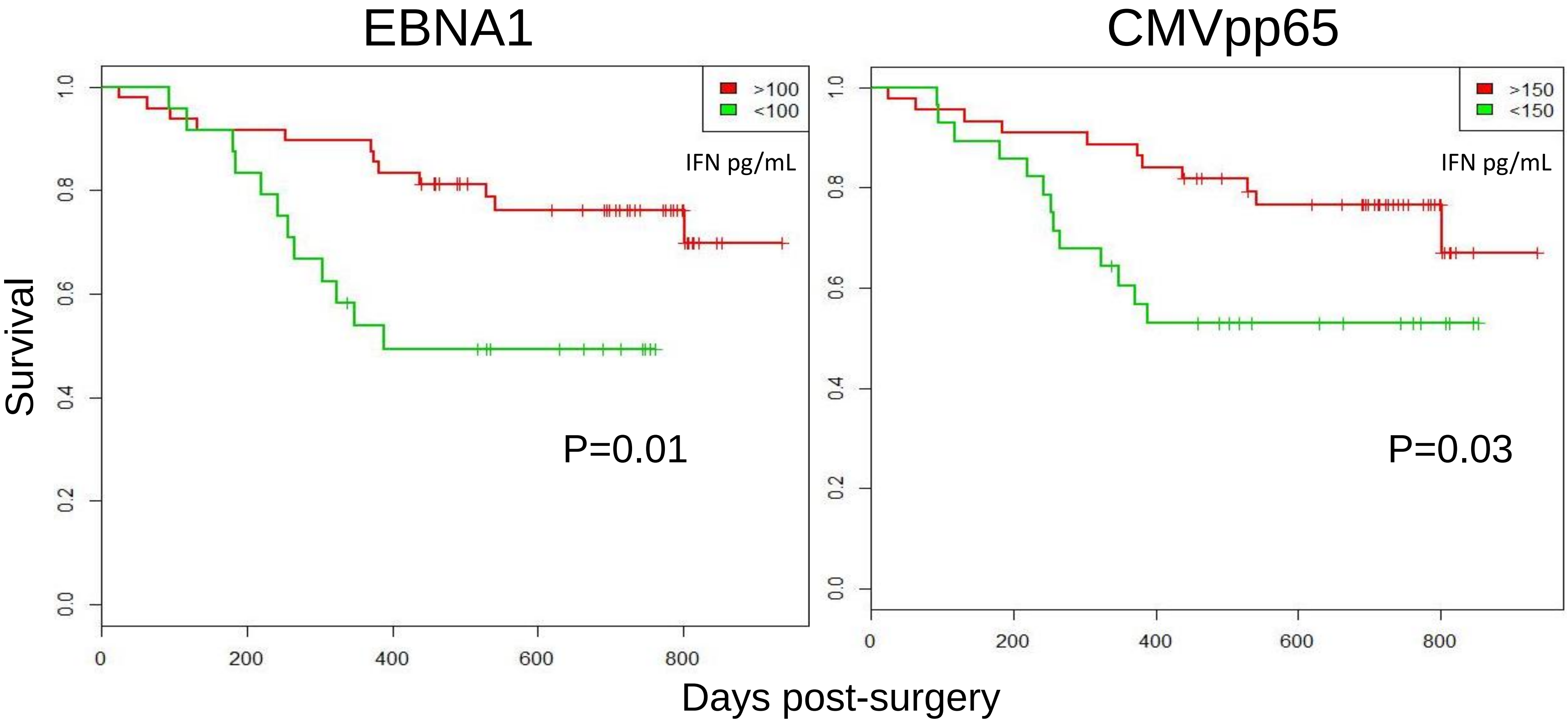

Supplementary Figure 2. The anti-EBV/CMV cellular immune response is associated with increased survival in patients with pancreatic cancer after surgery. Patients with pancreatic cancer who underwent surgery and consented to their tumour sample being used for translational and clinical research in accordance with the applicable ethical permit were clinically followed up for at least 800 days after the sampling time point (day of surgery). At the same juncture, venous blood was also drawn for immunoassays as described in the Materials of Methods section of this paper (whole blood assays with an antigen panel). A Kaplan-Meyer survival analysis was performed based on the following cut-off points for the concentrtion of IFN-γmeasured by ELISA after 7 days of culture: more than 100 pg/ml of cytokine indicated ‘strong’ cellular immune responses while less than 100 pg/ml of cytokine indicated ‘weak’ cellular immune responses. Patients with pancreatic cancer who mounted strong IFN-γ responses following exposure to EBNA-1 (p=0.01) and CMVpp65 (p=0.03) were also those who survived longer than patients who exhibited only weak anti-viral IFN-γ production.

Supplementary Figure S3

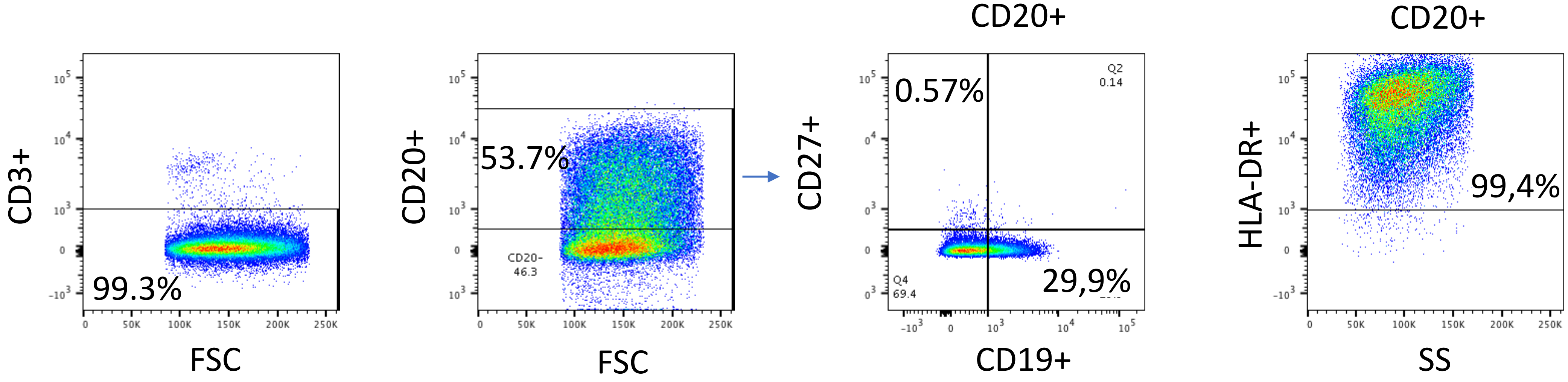

Supplementary Figure 3. EBV-immortalized tumor – infiltrating B-cells (TIB) from patient PanTT24 were negatively gated on CD3; CD3neg cells were gated on forward scatter versus CD20, which were positively gated to show CD19 versus CD27. The majoority of TIB were CD20+, CD27-, HLA-DR+ B-cells. *FSC: Forward scatter, SS: side scatter*
